# Electromagnetic Linear Vibration Energy Harvester Using Sliding Permanent Magnet Array and Ferrofluid as a Lubricant

**DOI:** 10.3390/mi8100288

**Published:** 2017-09-22

**Authors:** Song Hee Chae, Suna Ju, Yunhee Choi, Ye-Eun Chi, Chang-Hyeon Ji

**Affiliations:** Department of Electronic and Electrical Engineering, Ewha Womans University, Seoul 03760, Korea; pinekibo@naver.com (S.H.C.); suna5290@gmail.com (S.J.); younie89@naver.com (Y.C.); yeunchi@gmail.com (Y.-E.C.)

**Keywords:** energy harvester, ferrofluid, electromagnetic generator, low frequency vibration

## Abstract

We present an electromagnetic linear vibration energy harvester with an array of rectangular permanent magnets as a springless proof mass. Instead of supporting the magnet assembly with spring element, ferrofluid has been used as a lubricating material. When external vibration is applied laterally to the harvester, magnet assembly slides back and forth on the channel with reduced friction and wear due to ferrofluid, which significantly improves the long-term reliability of the device. Electric power is generated across an array of copper windings formed at the bottom of the aluminum housing. A proof-of-concept harvester has been fabricated and tested with a vibration exciter at various input frequencies and accelerations. For the device where 5 μL of ferrofluid was used for lubrication, maximum output power of 493 μW has been generated, which was 4.37% higher than that without ferrofluid. Long-term reliability improvement due to ferrofluid lubrication has also been verified. For the device with ferrofluid, 1.02% decrease of output power has been observed, in contrast to 59.73% decrease of output power without ferrofluid after 93,600 cycles.

## 1. Introduction

Recently, extension of battery lifetime has become a major concern for various applications. Harvesting energy from the environment has been widely researched as an alternative approach to overcome the technological difficulties and high cost in replacing primary batteries used in certain applications. Wireless sensor nodes, medical implants, and low power consuming wearable electronics are good examples. Among various sources of energy in nature, environmental vibration has attracted a substantial amount of attention from researchers due to advantages in accessibility and minimal constraints in utilization, abundance in the environment, and availability of straightforward power generation mechanisms.

Harvesting energy from vibration can be roughly classified based on three widely used transduction mechanisms: piezoelectric, electromagnetic, and electrostatic mechanisms [1,2,3,4,5,6,7,8]. A piezoelectric energy harvester generates electric energy by internal generation of electric charge when time-varying strain is induced in a piezoelectric material. Vibration applied to a movable part of a capacitor in the electrostatic energy harvester induces variation of capacitance and generates power with the aid of a polarization source. An electromagnetic energy harvester utilizes the change of magnetic flux linking the coils to induce electric current. Electromagnetic energy harvesting is an attractive candidate for vibration energy harvesters due to a rather straightforward mechanism of converting relative motion between magnet and coil into electric energy and ability to provide relatively high output power.

In the design of electromagnetic energy harvesters, utilization of a spring-mass-damper system in supporting the proof mass has been a common practice up to the present. External vibration applied to the device is transformed into relative motion between the permanent magnet and coil by supporting either the magnet or the coil using spring element. Despite the advantages of simple structure and relatively high efficiency at resonance, an energy harvester based on spring-mass-damper-based system inherently suffers from relatively high operation frequency and narrow bandwidth for certain applications. These properties make it difficult to utilize devices with spring-supported proof mass in harvesting energy from low frequency vibrations such as human-motion-induced vibration, which can be characterized by very low frequency, random and high amplitude motion. A springless system that can convert low frequency vibration to large displacement of proof mass without or with small frequency dependence comes to the fore as an alternative. In a springless-mass-based system, proof mass is not suspended by a spring element and can move freely in a confined pathway or cavity in which proof mass can repeat regular motion under the external vibration.

For conventional macro scale power generators, the electromagnetic power generation principle and rotary turbines supported by ball bearings dominated. For miniature and micro scale power generators, rotary turbines supported by miniature bearings, metal balls, and air-bearing have been proposed [9,10,11]. In contrast, spherically or cylindrically-shaped permanent magnets or metal structures have been utilized as springless proof mass in vibration energy harvesters to provide smooth motion with less friction and wear [12,13,14,15,16,17,18,19]. Bowers et al. utilized a spherical permanent magnet rolling randomly inside the cavity wrapped with coil [12] and Pillatsch et al. developed an impulse-excited harvester where a cylindrical proof mass actuates an array of piezoelectric bimorph beams through magnetic attraction [14]. Roundy et al. developed a piezoelectric harvester for a tire pressure monitoring application where spherical proof mass rolling inside a track indirectly pushes the piezoelectric beams [16]. Ju et al. proposed a magnetoelectric harvester where a spherical permanent magnet actuates the magnetoelectric laminate composite and impact-based piezoelectric harvester using a metal ball [17,18]. Among the previously reported harvesters using rolling motion of springless proof mass, random [12,13,15] or linear motion [14,16,17,18] of the proof mass have been utilized. Utilization of spherically or cylindrically-shaped proof mass provides a simple way of generating frequency-independent large displacement motion in harvesting devices, but effective use of magnetic flux with narrow gap between magnet and coil becomes very challenging for electromagnetic harvesters. Although a semicircular rotor supported by a ball bearing has been utilized in the development of a piezoelectric harvesting device [19], utilization of mechanical bearings has not been a general practice in the development of vibration energy harvesters due to limitations in device miniaturization, direction of the external vibration and low input acceleration.

**Figure 1:**
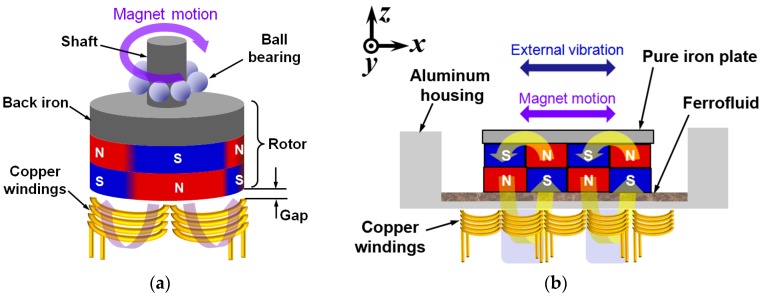
Comparison of electromagnetic power generator structures: (**a**) axial-flux rotary power generator; (**b**) linear vibration energy harvester.

This paper presents an electromagnetic vibration energy harvester using linear motion of springless proof mass. An array of rectangular magnets oscillates laterally without conventional spring support under the external vibration, providing a time-varying magnetic field to an array of coils located under the channel. Ferrofluid has been used as a lubricating material between the magnets and the underlying channel. The proposed architecture utilizes that of the axial flux rotary power generators as shown in Figure 1 [10]. Although rotational motion is favorable for conventional power generation devices and systems, linear motion can be advantageous for some of the energy harvesting applications due to the inherent nature of the external vibration used as an energy source. In general, the rotor of an axial flux generator is connected to the shaft supported by the ball bearing, which maintains the narrow gap between the magnets and copper windings to maximize the output power (Figure 1a). The proposed design utilizes a linear motion instead of the rotational counterpart to make use of the linear external vibration and to provide simple structure (Figure 1b). The minimum gap between the winding and magnet array is defined by the thickness of the bottom side of the channel. By lubricating the channel with ferrofluid, friction and wear at the magnet and channel interface can be reduced. Moreover, the requirement for a precise control of the gap between the rotor and winding can be obviated, which greatly simplifies the fabrication process.

In this research, we have designed, fabricated and tested a proof-of-concept electromagnetic vibration energy harvester using ferrofluid as a lubricant. The implemented energy harvester has been tested with a vibration exciter in various conditions and output characteristics of the device have been analyzed. Effects of the channel surface topography and ferrofluid droplet size have also been analyzed.

## 2. Harvester Design

**Figure 2:**
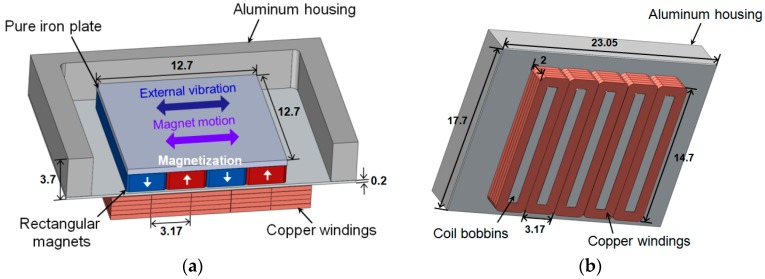
Schematic diagram of the proposed electromagnetic energy harvester (unit: mm): (**a**) top side; (**b**) bottom side.

A schematic diagram of the proposed energy harvester is shown in Figure 2. The proof-of-concept harvester consists of two parts: a freely sliding magnet assembly and aluminum housing with fixed coils. A multi-pole magnet array composed of four bar magnets and a pole piece serves as a proof mass moving inside the channel in response to external vibration. The pole piece covers the top side of the magnets, thereby enhancing the magnetic flux linking the coils placed under the aluminum housing. An array of copper windings is fixed at five coil bobbins and connected in series. Copper winding has a pitch of 3.17 mm, which matches the pitch (width) of the magnet. A minimum gap between the magnet array and coil is 0.2 mm, which is the minimum thickness of the aluminum housing. As the proof mass is not supported by a spring element, magnet assembly makes constant impacts with the sidewall of the aluminum housing when external vibration is applied.

The proof mass (magnet assembly) can move freely inside the channel with the aid of ferrofluid as a lubricating material at the interface between the bottom of the magnet array and the channel. Ferrofluid is a colloidal suspension containing ferromagnetic nanoparticles, which is strongly magnetized in the presence of a magnetic field. It is possible to use ferrofluid as a lubricant for permanent magnets without substantial decrease in magnetic field intensity, as the ferrofluid itself has larger than unity magnetic permeability and forms a very thin layer under the magnet array. Due to the inherent nature of gathering around regions of higher magnetic flux density, agglomerations are formed along the edges of the magnets when a droplet of ferrofluid is dispensed on the surface of magnet array, which is squeezed to form a thin layer when the magnet array is placed on the channel. Therefore, magnets tend to float on a thin layer of ferrofluid, sliding in the channel with reduced friction and wear in response to external vibration. When the external vibration is applied laterally to the harvester, magnet motion induces the variation of magnetic flux through the copper winding, which, in turn, generates electric power.

Proposed device has been designed in consideration of the following factors: (1) simplicity of the structure, (2) ease of fabrication process, and (3) low profile and small volume of the device. Although not covered in this work, for further miniaturization of the device using microfabrication, a planar coil geometry under the permanent magnet assembly could be beneficial considering the complexity of the multi-layer coil fabrication process.

## 3. Operation Principle

### 3.1. Output Voltage

As the time-varying magnetic field applied to the coil is induced by lateral motion of the magnet assembly, generated voltage is equal to the negative of local change in the magnetic flux (Φ) through the coils. For an ideal case where magnet assembly is traveling back and forth in the *x*-direction as shown in Figure 1b, time variation of magnetic flux density integrated along the *x*-axis is required to obtain the output voltage [3].

To obtain an analytic model of the magnetic field distribution, sinusoidal distribution along the *x*-axis and exponential decay in the *z*-axis have been assumed. Vertical component of the time-varying magnetic flux (*B_z_*) can be obtained by combining the magnet motion with magnetic field distribution obtained by finite element analysis (FEA). Magnetic flux density *B_z_*(*x*’) of the moving magnet array can be described by multiplication of exponential decay function in a vertical (*z*) direction and sinusoidal variation in a lateral (*x*) direction, as shown in Equation (1):

(1) $$B_{z}\left(x\prime \right)=\;B_{max}e^{-\alpha z\left(i\right)}\sin\left(\frac{2\pi }{p}\left(x\prime -x\left(t\right)\right)\right),$$

where *B_max_* is the maximum value of the magnetic flux density along the *z*-axis, *α* is the exponential decay constant and *z*(*i*) is the distance from *i*th turn of the coil to the magnet array, *p* is double the magnet pitch, *x*(*t*) is the displacement of the magnet array and *x’*(*t*) is the differential value along time of *x*(*t*) [3].

**Figure 3:**
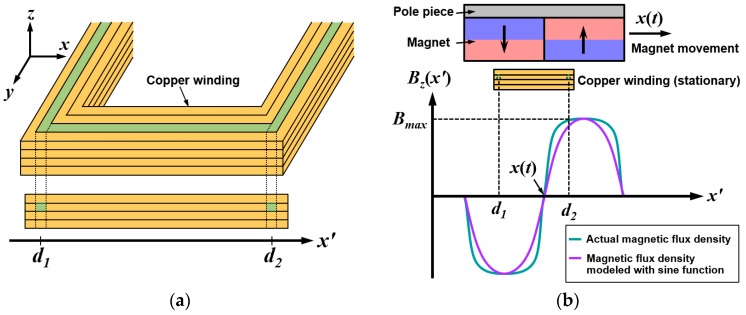
(**a**) Schematic diagram of the position of the coil turns in copper winding; (**b**) magnetic flux density distribution induced by lateral motion of the magnets [3].

After obtaining the magnetic flux by integrating the magnetic flux density *B_z_*(*x*’) along the *x*’-axis, open circuit voltage of the device can be calculated (dΦ/dt). The result is shown in Equation (2):

(2) $$\begin{array}{l} V=NlB_{max}e^{-\alpha z\left(i\right)}x^{\prime }\left(t\right)\{\lfloor \sin\left(\frac{2\pi d_{2}}{p}\right)\\ \;-\sin\left(\frac{2\pi d_{1}}{p}\right)\rfloor \cos\left(\frac{2\pi }{p}x\left(t\right)\right)\lfloor -\cos\left(\frac{2\pi d_{2}}{p}\right)\\ \;-\cos\left(\frac{2\pi d_{1}}{p}\right)\rfloor \sin\left(\frac{2\pi }{p}x\left(t\right)\right)\;\}, \end{array}$$

where *V* is the generated open circuit voltage, *N* is the number of turns of the coil, and *l* is the length of the coil along the direction perpendicular to the moving direction of the magnet [3]. As shown in Figure 3a, *d*_1_ and *d*_2_ denote the position of the coil turns in the *x*’-axis. Figure 3b shows two consecutive magnets, a cross section of the copper winding, and sinusoidally distributed *B_z_*(*x*’). Due to movement of the magnet assembly, *x*(*t*) is moving along the *x*’-axis in time, but *d*_1_ and *d*_2_ are not changed as copper winding is stationary.

### 3.2. Magnetic Flux Density at Different Gaps between the Coil and Magnet

**Figure 4:**
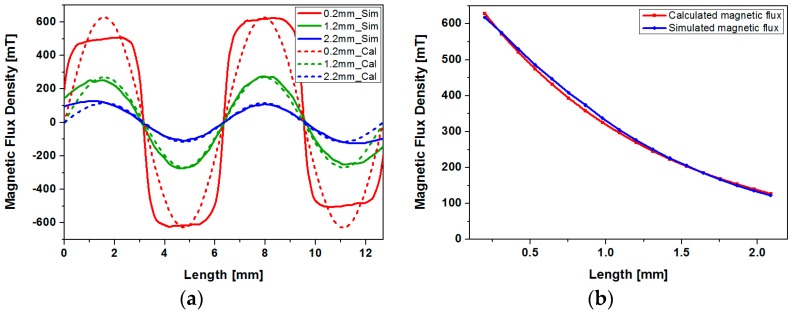
Simulation and curve fitting results of the magnetic flux density at various gaps from the magnet: (**a**) magnetic flux along the *x*-axis at various gaps (solid line shows the simulation result and dashed line shows the magnetic field distribution represented with sine function); (**b**) maximum magnetic flux density at various gaps.

The gap between the magnet assembly and coils significantly affects the magnetic flux density at the coil region. The copper winding has 2 mm-height in addition to a minimum of a 0.2 mm-gap, which coincides with the thickness of the channel floor. A two-dimensional FEA tool (FEMM, Finite Element Method Magnetics) has been used to obtain the distribution of magnetic flux density in the *x*- and *z*-directions. Solid lines in Figure 4a show the simulated vertical magnetic flux density distribution on top of an array of four magnets at various gaps. To obtain the sine distribution of magnetic flux density along the *x*-axis, a half period of the sine function has been matched with individual permanent magnet width, and amplitude has been chosen to match the maximum value of the simulated magnetic flux density. To obtain the magnetic flux density at different gaps along the *z*-axis, the maximum values of the simulated magnetic flux density at different gaps have been sorted out, and have been fitted with exponential decay function as shown in Figure 4b. Obtained maximum value of the magnetic flux density along the *z*-axis (*B_max_*) and exponential decay constant (α) in Equation (1) are 0.805 and 0.875, respectively. Dashed lines in Figure 4a shows the magnetic field distribution modeled with simple sine function with exponential decay. Although actual distribution of the magnetic flux (solid line in Figure 4a) deviates from the sine function, flux distribution can be modeled with moderate error. As shown in Figure 4a, discrepancies between the simulated magnetic flux density and sine representation of the flux density used in modeling increases as the gap between the magnet and coil is reduced. In addition, error increases as the distance from the center of the magnet array is increased.

## 4. Fabrication

**Figure 5:**
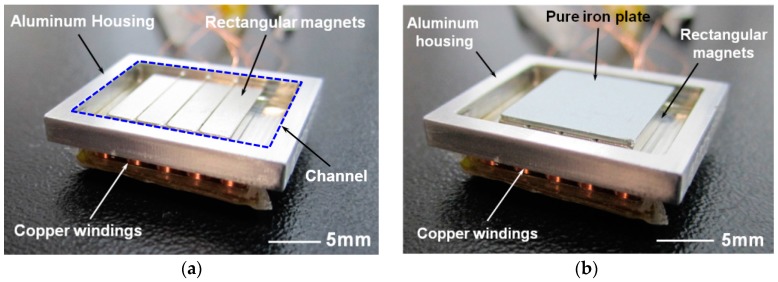
Proof-of-concept energy harvester after assembly: (**a**) pole piece covering the magnets and acrylic plate on top has been removed for visibility of the magnet array; (**b**) with pole piece covering the magnet array.

A proof-of-concept electromagnetic energy harvester has been fabricated with aluminum housing, bar-shaped NdFeB (Neodymium Iron Boron) magnets (N42 grade, K&J Magnetics Inc., Pipersville, PA, USA) and copper windings. Aluminum housing has been fabricated by a precision CNC (Computer Numerical Control) milling process (Figure 5). Each magnet measures 3.18 × 12.7 × 1.59 mm^3^ and magnet array consisting of four magnets is covered with a pole piece made of pure iron, which measures 12.7 × 12.7 × 0.2 mm^3^. Length and width of the channel are 19.05 mm and 14.7 mm, respectively. Thickness of the bottom side of the channel, which defines the minimum gap between the permanent magnets and copper windings, is 0.2 mm. A set of five coils have been fabricated using copper wire with 0.1 mm diameter and has been connected in series. After comparing the effect of total winding height on output power, self-supporting coils 2 mm in height and 140 turns have been fabricated. Figure 4 shows the assembled energy harvester, with and without a pole piece. The assembled device, which measures 23.05 × 18.7 × 4.5 mm^3^, has been covered with an acrylic plate to prevent the magnet array from bouncing off the housing during the experiments.

**Figure 6:**
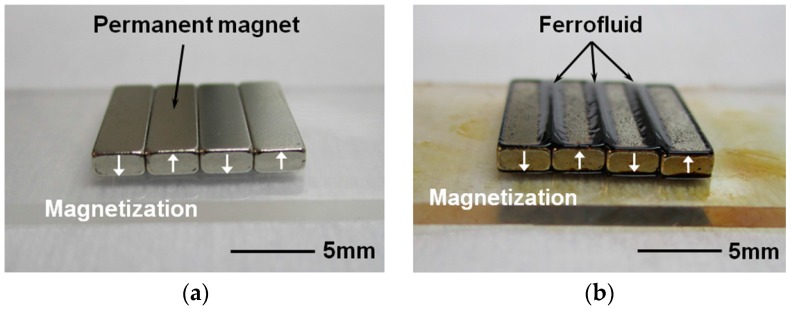
Magnet array: (**a**) in pristine condition; (**b**) after ferrofluid droplet dispense.

Output voltage and power of the fabricated energy harvester have been tested with and without ferrofluid (Apex Magnets, Petersburg, WV, USA) to verify its effectiveness as a lubricant. When the ferrofluid droplet is dispensed on the magnet surface, ferrofluid tends to gather at the magnet edges where the magnetic flux is higher as shown in Figure 6b. Agglomerated ferrofluid is squeezed to form a thin layer at the interface between magnet array and bottom surface of the channel, which tends to follow the magnet motion during operation. As discussed in the latter section, only a small amount of ferrofluid is required to reduce the friction and wear without concerns about leakage and increased gap between magnet and coils.

## 5. Experimental Results and Discussion

**Figure 7:**
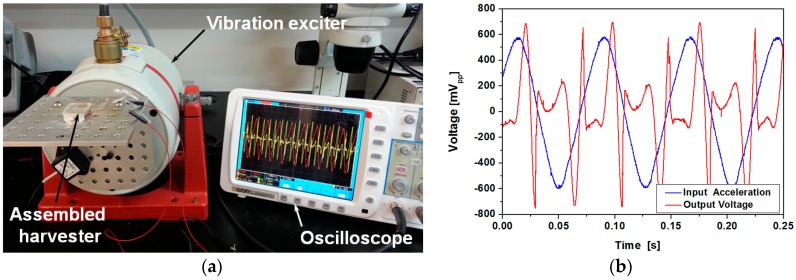
(**a**) Experimental setup for the vibration exciter test; (**b**) input acceleration and open-circuit voltage of the device with 5 uL ferrofluid (3 g acceleration at 13 Hz applied).

Fabricated energy harvester has been driven by a vibration exciter (LDS 406, Brüel & Kjær, Nærum, Denmark) and output from the device has been observed with an oscilloscope. Input frequency has been increased from 7 to 20 Hz with 1 Hz step and acceleration has been increased from 1 to 3 g with 1 g step. Vibrations at frequency lower than 6 Hz and acceleration higher than 3 g could not be applied due to the limitation of the test equipment. Figure 7a shows the experimental setup for the vibration exciter test. The assembled device has been mounted horizontally on the vibration exciter and sinusoidal input vibration is laterally applied to the harvester. The device generates output voltage in response to input acceleration as shown in Figure 7b.

To our knowledge, this is the first attempt to utilize ferrofluid as a lubricant for the magnetic springless proof mass in a vibration energy harvester. For a better understanding of the device performance and effect of utilizing ferrofluid as a lubricant, open circuit voltage waveform has been compared with that of the analytic model. Moreover, further experiments have been performed to analyze the effect of channel surface roughness and ferrofluid droplet size. Each experiment has been carried out with and without ferrofluid. Lastly, long-term reliability has been analyzed by cyclic testing with and without ferrofluid.

### 5.1. Comparison between Estimated Output Voltage and Experiment Results

**Figure 8:**
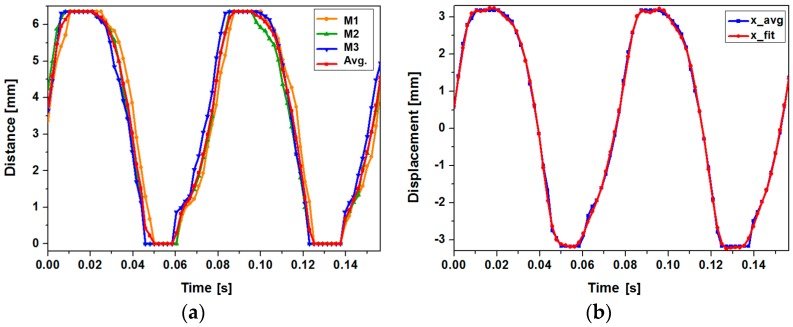
Motion of the magnet array in the harvester under the 3 g acceleration in 13 Hz: (**a**) experimentally determined position of the magnet array (M1–M3: individual measurements, Avg.: average of the measurements); (**b**) curve-fitting result of the magnet array position.

We have analyzed the open circuit voltage of the device using the theoretical model provided in the previous section. As the magnet array undergoes a non-sinusoidal motion even at the presence of sinusoidal vibration, motion of the magnet array has been analyzed experimentally. Motion of the magnet array with ferrofluid under 3 g acceleration at 13 Hz has been captured with a video camera at 480 frames per second. Total travel range of the magnet array in a lateral direction is approximately 6.35 mm. Although the magnet array collides with both ends of the channel during operation, actual motion of the magnet mimics a sinusoidal form with slight distortion. Figure 8a shows the individual and averaged results of the magnet position measurement. Average displacement of the magnet array has been curve-fitted with MATLAB (R2016a, The MathWorks, Inc., Natick, MA, USA) as shown in Figure 8b. The displacement can be described by a sum of eight sine functions with negligible error. The fitted function for displacement *x*(*t*) can be expressed with Equation (3):

(3) x(t)=3.503sin(80.79t+0.1797)+9.251sin(21.05t−1.327)+0.352sin(243.5t−0.3331)+3.054sin(172.4t+1.155)+9.023sin(22.24t+1.71)+0.1031sin(405.7t−1.05)+0.06929sin(561t−2.175)+2.877sin(173.1t+4.206).

**Figure 9:**
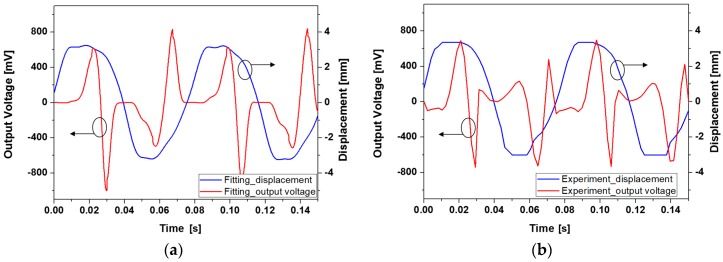
Comparison of the open circuit voltage waveforms: (**a**) calculated output voltage and displacement; (**b**) experimented output voltage and displacement.

By combining Equations (1) and (3), open circuit voltage of the harvester can be obtained. Figure 9 shows the waveforms of estimated and experimented open circuit voltages. Experiment has been carried out at input frequency of 13 Hz and 3 g acceleration with 5 µL of ferrofluid. Although amplitude of individual peaks do not match perfectly, the overall shape and trend of the output voltage waveform is in relatively good agreement with the predicted counterpart. Discrepancies between the two waveforms can be ascribed to the non-ideal movement of the magnet array due to friction and unwanted motions in directions other than the *x*-direction, gap between the magnet and coil, and approximations in magnetic field distribution. As the output voltage is proportional to the variation of magnetic flux linking each coil, displacement of the magnet assembly critically affects the output voltage waveform. When the displacement of the magnet is not smooth enough to be expressed with the combination of sine functions shown in Equation (3), resulting output voltage waveform deviates more from the measurement result. This type of deviation is well-represented in time segments between 0.45 s to 0.9 s in Figure 9a,b. Estimated and measured maximum peak-to-peak open circuit voltage are 1.616 V and 1.432 V, respectively.

### 5.2. Effect of the Channel Surface Roughness

**Figure 10:**
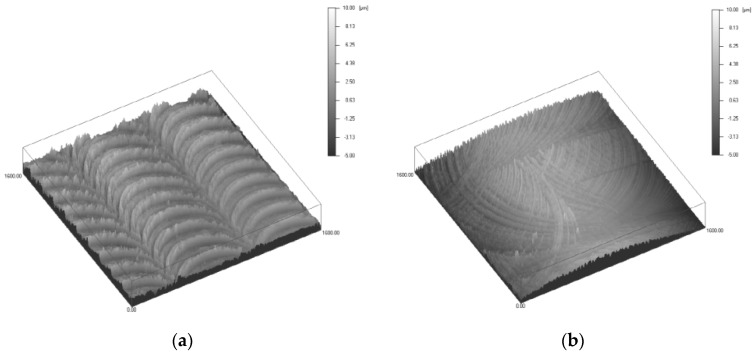
3D profile for surface roughness of two types of housing channel (**a**) type 1; (**b**) type 2.

Due to direct contact between magnet array and channel, motion of the magnet array can be affected by roughness of the channel surface, which also affects the output characteristics. Two types of aluminum channels with different average surface roughness have been prepared and the effect of roughness has been analyzed experimentally. Figure 10 shows the 3D profiles of the two types of channels measured with confocal microscope (μsurf, NanoFocus, Oberhausen, Germany). As shown in Figure 10 and Table 1, type 1 has a rougher surface with an average roughness of 1044 nm compared to that of 664 nm for type 2.

**Figure 11:**
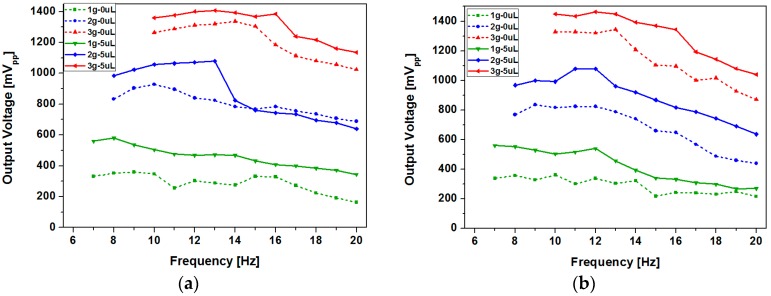
Peak-to-peak open circuit voltage at various input frequencies and accelerations when channels of different surface roughness are used: (**a**) type 1; (**b**) type 2.

To analyze the effect of surface roughness and effectiveness of ferrofluid lubrication, we have measured and compared the peak-to-peak open circuit voltages of the devices with two different channel surfaces at various input frequencies and accelerations. Both devices have been tested with 5 µL of ferrofluid and without ferrofluid. As shown in Figure 11, open circuit voltage showed an increasing trend as the acceleration was increased for both devices. Improvement of open circuit voltage due to the use of ferrofluid was more pronounced in the type 2 device, which had smaller surface roughness (Figure 11b). In contrast, the type 1 device showed small improvement or even deterioration at some points (Figure 11a). For the type 1 device, maximum open circuit voltage of 1.34 V has been obtained at 3 g acceleration at 14 Hz without ferrofluid, and 1.41 V has been achieved at 3 g acceleration at 13 Hz with 5 µL of ferrofluid. For the type 2 device, maximum open circuit voltage of 1.34 V has been generated at 3 g acceleration at 13 Hz without ferrofluid, and 1.46 V has been achieved at 12 Hz and 3 g acceleration with 5 µL of ferrofluid.

**Figure 12:**
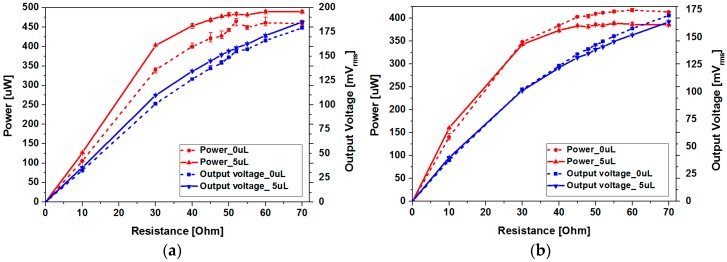
Output power and voltage at various load resistances for devices with different channel surface roughness: (**a**) type 1; (**b**) type 2.

**Figure 13:**
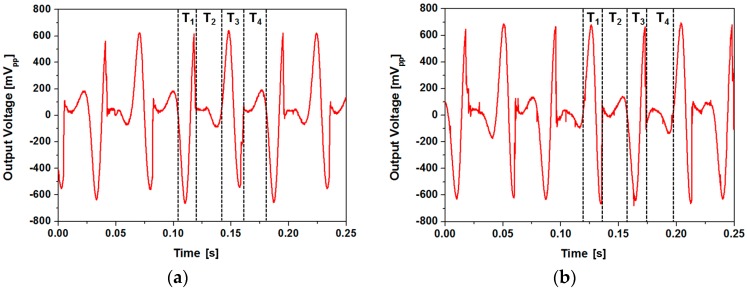
Open circuit voltage waveforms (devices with ferrofluid): (**a**) type 1; (**b**) type 2.

Figure 12 shows the output power and root mean square (RMS) voltage of the two devices with and without ferrofluid. For the type 1 device, maximum output power of 472.35 μW has been achieved at load resistance of 52 Ω without ferrofluid, while 493 μW has been obtained at load resistance of 60 Ω with ferrofluid. The type 2 device has generated maximum output power of 421 μW at load resistance of 60 Ω without ferrofluid and 389.13 μW at load resistance of 55 Ω with ferrofluid. Despite the increase in peak-to-peak open circuit voltage after addition of ferrofluid for both devices, output power decrease has been observed in the type 2 device. Differences in output power change due to ferrofluid addition can be explained with output voltage waveforms (Figure 12). Output power (*P*) was determined using the following equation:

(4) $$P=\frac{1}{T}\int_{0}^{T}\frac{v{\left(t\right)}^{2}}{R}dt=\frac{V_{rms}}{R},$$

where *v*(*t*), *T*, *R* and *V_rms_* are the instantaneous output voltage, period, load resistance and root-mean-square voltage, respectively. As the output power not only depends on the amplitude of the voltage signal but also on the time duration of individual peak, time spent while the magnet assembly contacts the channel end does not contribute to output power generation. As shown in Figure 13, ratio of the time spent during power generation (*T*_1_ + *T*_3_) during each cycle was 49.8% for the type 1 device and 43.4% for the type 2 device. As the moving speed of the magnet was faster for the type 2 device, amplitude of the voltage signal was higher, but more time was spent while the magnet contacts the channel end. Proposed device requires further optimization to take surface morphology and hydrophobicity of the channel surface into account. We have used the type 1 device with a rougher surface for other experiments to further improve the performance of the device.

### 5.3. Effect of Ferrofluid Droplet Size

**Figure 14:**
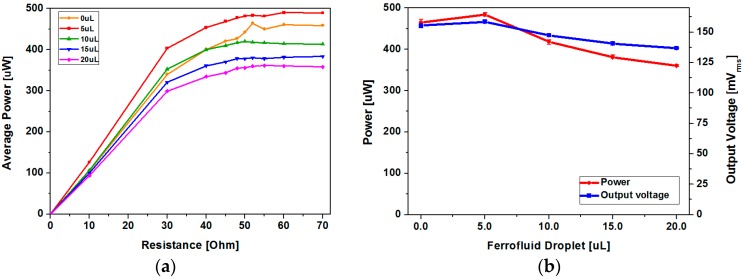
Comparison of the effect of ferrofluid droplet size (3 g acceleration at 13 Hz applied): (**a**) average power at various load resistances; (**b**) power and output RMS voltage at 60 Ω load resistance.

Although it is clear from the experiment that ferrofluid forms a thin layer between the magnet array and the channel, lubrication regime, or the type of lubrication, determined by the thickness of the ferrofluid film is unclear. As the applicable acceleration from the environment and thus the speed of magnet movement is limited, we have simply tried to analyze the effect of the dispensed ferrofluid droplet size on generated output power to optimize the device performance. When the amount of dispensed ferrofluid is excessive, magnetic flux density at the coil region becomes smaller as the gap between the magnet and coil increases, which results in a decrease of output voltage. In addition, when the amount of dispensed ferrofluid is too small, output voltage decreases due to insufficient or lack of lubrication. To characterize the effect of ferrofluid droplet size on output power, a vibration exciter test has been carried out with ferrofluid droplet size ranging from 0 to 20 μL with 5 μL step. As shown in Figure 14, maximum power has been obtained at a minimum droplet size of 5 μL. Decrease of output power with increasing ferrofluid droplet size can be ascribed to the change in lubrication regime and increased gap between the magnet and coil. Maximum average power of 489.95 μW has been achieved with 5 μL of ferrofluid at a load resistance of 60 Ω, which drops to 361.49 μW when the droplet size is increased to 20 μL.

### 5.4. Reliability Improvement Using Ferrofluid as a Lubricant

**Figure 15:**
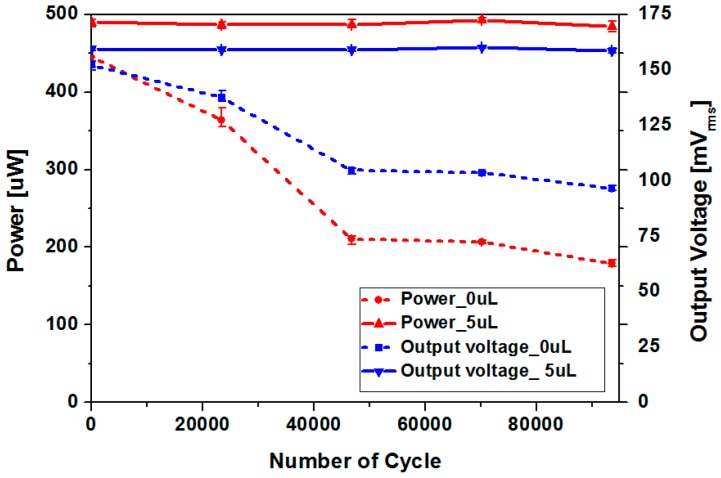
Output power and voltage variation during cyclic testing (input acceleration: 3 g, input frequency: 13 Hz).

**Figure 16:**
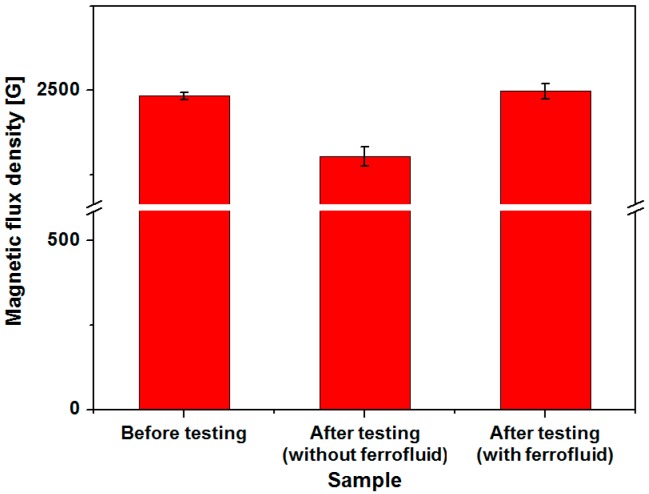
Magnetic flux variation of the permanent magnet before and after cyclic testing.

In order to verify the effectiveness of ferrofluid in terms of long-term reliability, output voltage and power of the harvesters with and without ferrofluid have been compared after cyclic testing. Although output power increase of the harvester due to addition of ferrofluid was approximately 4%, the original device without lubrication is subject to non-negligible in-use degradation as the magnets are directly contacting the channel surface. Sinusoidal input acceleration of 3 g at 13 Hz has been applied with a vibration exciter. As shown in Figure 15, output power of the device without ferrofluid has been reduced from 445.22 μW to 179.27 μW after 93,600 cycles of magnet oscillation. Approximately 59.73% decrease in generated power has been observed with the device without ferrofluid. Substantial decrease of output power has already been observed after 20,000 cycles, which suggests that the degradation has been initiated at the early phase of cyclic testing. Reduction of output power has started to saturate after 40,000 cycles. In contrast, a harvester with 5 μL of ferrofluid showed unnoticeable or very little change in output power and voltage. Output power has been changed from 489.62 μW to 484.61 μW after 93,600 cycles, with only 1.02% drop. In addition, magnetic flux density of the permanent magnets has been measured and compared before and after testing. As shown in Figure 16, average value of the magnetic flux density at magnet surface was 2483 G for magnets in pristine condition. In contrast to magnets used in cyclic testing without ferrofluid whose magnetic flux density dropped to 2304 G, average flux density of 2497 G has been measured with magnets tested with ferrofluid lubrication. Considering the measurement error, no deterioration of magnet strength has been observed for device with ferrofluid. Although output power improvement due to addition of ferrofluid was small, substantial enhancement in long-term reliability has been observed, which verifies the use of ferrofluid as a lubricant. In addition to the contact between the magnet and bottom surface of the channel, the magnet array constantly collides with both ends of the channel. From the experimental results shown in Figure 15, it can be concluded that the magnet degradation due to collision is negligible under applied testing conditions.

### 5.5. Comparison of Ouput Power

Overall performance of the proposed linear vibration energy harvester has been compared with previously reported devices. Table 2 shows a summary of the power generation performances of various non-resonant type vibration energy harvesters. Except for the electromagnetic device with randomly moving spherical permanent magnet [13], all the devices in the table utilized linear motion of the proof mass. Although generated power is smaller and input acceleration is larger than some of the previously reported devices, relatively high output power density and power density per input acceleration have been achieved within less than 2 cm^3^ volume of the developed device. Further development of an electromechanical model that includes the effect of impact and friction could potentially provide a more analytic approach to the proposed concept and optimized device geometry with improved performance.

## 6. Conclusions

We have proposed an electromagnetic vibration energy harvester to generate power from low frequency input vibration such as human-body-induced motion using a springless permanent magnet and ferrofluid as a lubricant. A proof-of-concept vibration energy harvester has been designed, fabricated, and tested. An analytical model of the device has been proposed and theoretical output voltage has been compared with the experimental results. Moreover, effects of ferrofluid droplet size and roughness of the channel surface have been analyzed by experiments. Maximum output power of 493 μW has been generated using 5 μL of ferrofluid as a lubricant. The effect of ferrofluid in long-term reliability of the proposed energy harvester has been verified by cyclic testing, where only 1.02% of output power decrease has been observed after 93,600 cycles of operation, compared to 59.73% decrease of power in devices without ferrofluid. Although further optimization could lead to additional increase in output power, we have successfully demonstrated the feasibility of utilizing ferrofluid as a lubricating material for a springless electromagnetic vibration energy harvester for low frequency vibrations.

## Figures and Tables

**Table 1:** Surface roughness of different types of channels.

Model	Type 1	Type 2
Average surface roughness (nm)	1044	644
Standard deviation of surface roughness (nm)	1344	861

**Table 2:** Comparison of power generation performances of various harvesting devices.

Reference	Power Generation Mechanism	Maximum Average Power (μW)	Frequency at Maximum Power (Hz)	Acceleration at Maximum Power (g)	Volume (mm^3^)	Power Density (μW/mm^3^)	Power Density/Acceleration (μW/mm^3^/g)
This work	Electromagnetic	493	13	3	1940	2.54 × 10^−1^	8.47 × 10^−2^
[2]	Piezoelectric	600 *	10	hand shake	14,000	4.29 × 10^−2^	-
[8]	Electromagnetic	795,000	4.6–14.5	0.17	1,361,800	5.84 × 10^−1^	3.43
[13]	Electromagnetic	300	ankle motion	ankle motion	100,000	3.00 × 10^−3^	-
[14]	Piezoelectric	2100	2	0.296	125,000	1.68 × 10^−2^	5.68 × 10^−2^
[18]	Piezoelectric	246	17	3	2076	1.18 × 10^−1^	3.95 × 10^−2^

* Maximum peak power.

## References

[1] Roundy S., Wright P.K. (2004). A piezoelectric vibration based generator for wireless electronics. Smart Mater. Struct..

[2] Renaud M., Fiorini P., van Schaijk R., van Hoof C. (2012). Harvesting energy from the motion of human limbs: The design and analysis of an impact-based piezoelectric generator. Smart Mater. Struct..

[3] Roundy S., Takahashi E. (2013). A planar electromagnetic energy harvesting transducer using a multi-pole magnetic plate. Sens. Actuators A Phys..

[4] Koukharenko E., Beeby S.P., Tudor M.J., White N.M., O’Donnell T., Saha C., Kulkarni S., Roy S. (2006). Microelectromechanical systems vibration powered electromagnetic generator for wireless sensor applications. Microsyst. Technol..

[5] Cheng S., Arnold D.P. (2010). A study of a multi-pole magnetic generator for low-frequency vibrational energy harvesting. J. Micromech. Microeng..

[6] Meninger S., Mur-Miranda J.O., Amirtharajah R., Chandrakasan A., Lang J.H. (2001). Vibration-to-electric energy conversion. IEEE Trans. Very Large Scale Integr. (VLSI) Syst..

[7] Basset P., Galayko D., Paracha A.M., Marty F., Dudka A., Bourouina T. (2009). A batch-fabricated and electret-free silicon electrostatic vibration energy harvester. J. Micromech. Microeng..

[8] Abed I., Kacem N., Bouhaddi N., Bouazizi M.L. (2016). Multi-modal vibration energy harvesting approach based on nonlinear oscillator arrays under magnetic levitation. Smart Mater. Struct..

[9] Hergert R.J., Hanrahan B., Ghodssi R., Holmes A.S. (2013). Performance of integrated retainer rings in silicon micro-turbines with thrust style micro-ball bearings. J. Micromech. Microeng..

[10] Herrault F., Ji C.H., Allen M.G. (2008). Ultraminiaturized high-speed permanent-magnet generators for milliwatt-level power generation. J. Microelectromech Syst..

[11] Herrault F., Yen B.C., Ji C.H., Spakovszky Z.S., Lang J.H., Allen M.G. (2010). Fabrication and performance of silicon-embedded permanent-magnet microgenerators. J. Microelectromech. Syst..

[12] Bowers B.J., Arnold D.P. (2009). Spherical, rolling magnet generators for passive energy harvesting from human motion. J. Micromech. Microeng..

[13] Rao Y., Cheng S., Arnold D.P. (2013). An energy harvesting system for passively generating power from human activities. J. Micromech. Microeng..

[14] Pillatsch P., Yeatman E.M., Holmes A.S. (2012). A scalable piezoelectric impulse-excited energy harvester for human body excitation. Smart Mater. Struct..

[15] Choi Y., Ju S., Chae S.H., Jun S., Ji C.H. (2015). Low-frequency vibration energy harvester using a spherical permanent magnet with controlled mass distribution. Smart Mater. Struct..

[16] Roundy S.J., Tola J. An energy harvester for rotating environments using offset pendulum dynamics. Proceedings of the 2013 Transducers & Eurosensors XXVII: The 17th International Conference on Solid-State Sensors, Actuators and Microsystems (TRANSDUCERS & EUROSENSORS XXVII).

[17] Ju S., Chae S.H., Choi Y., Lee S., Lee H.W., Ji C.H. (2013). A low frequency vibration energy harvester using magnetoelectric laminate composite. Smart Mater. Struct..

[18] Ju S., Chae S.H., Choi Y., Ji C.H. (2015). Macro fiber composite-based low frequency vibration energy harvester. Sens. Actuators A Phys..

[19] Pillatsch P., Yeatman E.M., Holmes A.S. (2013). Real world testing of a piezoelectric rotational energy harvester for human motion. J. Phys. Conf. Ser..

